# Supplementary material for: The role of migration networks in the development of Botswana’s generalized HIV epidemic
Source: eLife. 2023 Sep 4;12:e85435. doi: 10.7554/eLife.85435 (PMC10476964; doi:10.7554/eLife.85435)
Supplement: Supplementary file 1. — These tables present each district’s urban/rural classification, population size, total number of migrants (both within and between districts), within district migration intensity per hundred residents (WDMI), and population turnover per hundred residents. The urban/rural classes were city (C), town (T), predominantly urban (U), partially urban (PU), and predominantly rural (R). Population sizes are tabulated for residents for whom there was 1-year migration data. In 2010–2011, the Central Kgalagadi Game Reserve district is denoted CKGR. [file elife-85435-supp1.docx]

**Supplementary file 1a: Migration metrics by district, 1980-81.**

|  |  |  |  | Number of Migrants | | |  | Metric | |
| --- | --- | --- | --- | --- | --- | --- | --- | --- | --- |
| ID | District | Class | Population | Within | In | Out |  | WDMI | Turnover |
| 1 | Gaborone | C | 56,390 | 120 | 7,750 | 6,110 |  | 0.22 | 3.00 |
| 2 | Francistown | C | 31,090 | 50 | 4,730 | 3,880 |  | 0.17 | 2.81 |
| 3 | Lobatse | T | 18,110 | 0 | 1,940 | 2,260 |  | 0.00 | -1.74 |
| 4 | Selebi Phikwe | T | 27,210 | 50 | 4,080 | 3,850 |  | 0.19 | 0.85 |
| 5 | Orapa | T | 6,330 | 0 | 810 | 890 |  | 0.00 | -1.25 |
| 6 | Jwaneng | T | 5,120 | 0 | 2,310 | 710 |  | 0.00 | 45.45 |
| 10 | Southern | R | 103,190 | 7,120 | 3,330 | 3,390 |  | 6.90 | -0.06 |
| 11 | Barolong | R | 14,290 | 170 | 340 | 770 |  | 1.15 | -2.92 |
| 20 | South East | R | 30,880 | 330 | 2,000 | 1,000 |  | 1.10 | 3.35 |
| 30 | Kweneng | R | 109,890 | 5,970 | 2,010 | 2,450 |  | 5.41 | -0.40 |
| 40 | Kgatleng | R | 43,110 | 1,740 | 1,330 | 1,960 |  | 3.98 | -1.44 |
| 50 | Central Serowe | R | 93,260 | 5,240 | 3,350 | 4,480 |  | 5.55 | -1.20 |
| 51 | Central Mahalapye | R | 78,910 | 3,120 | 2,530 | 3,200 |  | 3.92 | -0.84 |
| 52 | Central Bobonong | R | 44,160 | 3,100 | 1,840 | 2,730 |  | 6.88 | -1.98 |
| 53 | Central Boteti | R | 24,620 | 1,940 | 1,200 | 1,050 |  | 7.93 | 0.61 |
| 54 | Central Tutume | R | 74,850 | 2,790 | 3,230 | 3,940 |  | 3.69 | -0.94 |
| 55 | Central Tuli Block | R | 2,740 | 20 | 530 | 0 |  | 0.90 | 23.98 |
| 60 | North East | R | 36,360 | 890 | 1,890 | 2,480 |  | 2.41 | -1.60 |
| 70 | Ngamiland South | R | 58,160 | 2,870 | 1,280 | 1,360 |  | 4.93 | -0.14 |
| 71 | Ngamiland North | R | 7,760 | 90 | 140 | 310 |  | 1.13 | -2.14 |
| 72 | Chobe | R | 7,390 | 480 | 370 | 380 |  | 6.49 | -0.14 |
| 73 | Okavango Delta | R | 1,020 | 20 | 150 | 20 |  | 2.25 | 14.61 |
| 80 | Ghanzi | R | 17,890 | 1,620 | 390 | 530 |  | 8.99 | -0.78 |
| 90 | Kgalagadi South | R | 14,910 | 690 | 450 | 420 |  | 4.64 | 0.20 |
| 91 | Kgalagadi North | R | 8,880 | 170 | 430 | 240 |  | 1.96 | 2.19 |
|  | Botswana |  | 916,520 | 38,590 | 48,410 | 48,410 |  | 4.21 | 0 |

**Supplementary file 1b: Migration metrics by district, 1990-91.**

|  |  |  |  | Number of Migrants | | |  | Metric | |
| --- | --- | --- | --- | --- | --- | --- | --- | --- | --- |
| ID | District | Class | Population | Within | In | Out |  | WDMI | Turnover |
| 1 | Gaborone | C | 137,680 | 550 | 15,350 | 14,620 |  | 0.40 | 0.53 |
| 2 | Francistown | C | 67,740 | 70 | 8,110 | 6,510 |  | 0.11 | 2.42 |
| 3 | Lobatse | T | 26,570 | 0 | 3,130 | 2,640 |  | 0.00 | 1.88 |
| 4 | Selebi Phikwe | T | 40,480 | 30 | 4,920 | 4,110 |  | 0.08 | 2.04 |
| 5 | Orapa | T | 8,120 | 0 | 900 | 1,080 |  | 0.00 | -2.17 |
| 6 | Jwaneng | T | 10,520 | 0 | 1,450 | 1,800 |  | 0.00 | -3.22 |
| 7 | Sowa | T | 1,640 | 0 | 780 | 410 |  | 0.00 | 29.13 |
| 10 | Southern | R | 125,400 | 5,200 | 5,020 | 6,460 |  | 4.10 | -1.14 |
| 11 | Barolong | R | 18,050 | 420 | 700 | 1,400 |  | 2.24 | -3.73 |
| 20 | South East | U | 42,320 | 900 | 3,280 | 2,150 |  | 2.18 | 2.74 |
| 31 | Kweneng East | PU | 140,810 | 6,650 | 8,060 | 6,480 |  | 4.78 | 1.13 |
| 32 | Kweneng West | R | 27,700 | 1,360 | 1,630 | 1,430 |  | 4.95 | 0.73 |
| 40 | Kgatleng | PU | 55,180 | 2,670 | 3,040 | 3,200 |  | 4.82 | -0.29 |
| 50 | Central Serowe | R | 126,810 | 7,050 | 7,350 | 6,900 |  | 5.58 | 0.36 |
| 51 | Central Mahalapye | R | 97,030 | 5,360 | 4,690 | 6,360 |  | 5.43 | -1.69 |
| 52 | Central Bobonong | R | 51,220 | 2,780 | 2,220 | 3,320 |  | 5.31 | -2.10 |
| 53 | Central Boteti | R | 37,630 | 2,190 | 1,980 | 1,770 |  | 5.85 | 0.56 |
| 54 | Central Tutume | R | 98,940 | 3,590 | 5,000 | 6,960 |  | 3.56 | -1.94 |
| 60 | North East | R | 41,470 | 1,040 | 2,830 | 3,290 |  | 2.48 | -1.10 |
| 70 | Ngamiland South | PU | 60,450 | 4,650 | 2,470 | 4,400 |  | 7.45 | -3.09 |
| 71 | Ngamiland North | R | 33,750 | 620 | 2,650 | 650 |  | 1.95 | 6.30 |
| 72 | Chobe | PU | 12,500 | 700 | 1,320 | 1,100 |  | 5.70 | 1.79 |
| 80 | Ghanzi | R | 26,110 | 1,970 | 820 | 810 |  | 7.55 | 0.04 |
| 90 | Kgalagadi South | R | 19,250 | 1,010 | 910 | 780 |  | 5.28 | 0.68 |
| 91 | Kgalagadi North | R | 10,970 | 410 | 740 | 720 |  | 3.74 | 0.18 |
|  | Botswana (Total) |  | 1,318,340 | 49,220 | 89,350 | 89,350 |  | 3.73 | 0 |

**Supplementary file 1c: Migration metrics by district, 2000-01.**

|  |  |  |  | Number of Migrants | | |  | Metric | |
| --- | --- | --- | --- | --- | --- | --- | --- | --- | --- |
| ID | District | Class | Population | Within | In | Out |  | WDMI | Turnover |
| 1 | Gaborone | C | 195,150 | 1,360 | 22,100 | 25,420 |  | 0.69 | -1.67 |
| 2 | Francistown | C | 83,900 | 220 | 9,060 | 9,620 |  | 0.26 | -0.66 |
| 3 | Lobatse | T | 31,510 | 170 | 3,660 | 4,120 |  | 0.53 | -1.44 |
| 4 | Selebi Phikwe | T | 50,420 | 110 | 5,420 | 5,630 |  | 0.22 | -0.41 |
| 5 | Orapa | T | 8,770 | 10 | 1,450 | 1,100 |  | 0.12 | 4.16 |
| 6 | Jwaneng | T | 15,360 | 70 | 2,870 | 2,330 |  | 0.47 | 3.64 |
| 7 | Sowa | T | 3,150 | 0 | 680 | 790 |  | 0.00 | -3.37 |
| 10 | Southern | R | 113,600 | 4,850 | 6,940 | 7,670 |  | 4.24 | -0.64 |
| 11 | Barolong | R | 45,450 | 1,310 | 3,310 | 3,070 |  | 2.90 | 0.53 |
| 12 | Ngwaketse West | U | 10,840 | 380 | 930 | 1,000 |  | 3.48 | -0.64 |
| 20 | South East | U | 59,670 | 690 | 6,030 | 3,680 |  | 1.20 | 4.10 |
| 31 | Kweneng East | U | 185,880 | 6,010 | 11,760 | 9,540 |  | 3.27 | 1.21 |
| 32 | Kweneng West | R | 39,110 | 2,040 | 2,220 | 2,540 |  | 5.17 | -0.81 |
| 40 | Kgatleng | PU | 73,460 | 2,270 | 4,250 | 4,090 |  | 3.10 | 0.22 |
| 50 | Central Serowe | PU | 151,830 | 5,750 | 9,830 | 10,780 |  | 3.76 | -0.62 |
| 51 | Central Mahalapye | PU | 110,010 | 4,560 | 6,250 | 6,980 |  | 4.12 | -0.66 |
| 52 | Central Bobonong | PU | 64,330 | 2,710 | 4,090 | 3,420 |  | 4.26 | 1.05 |
| 53 | Central Boteti | PU | 47,110 | 3,780 | 3,110 | 2,140 |  | 8.19 | 2.10 |
| 54 | Central Tutume | R | 121,520 | 4,390 | 6,630 | 8,030 |  | 3.57 | -1.14 |
| 60 | North East | R | 49,930 | 1,310 | 3,740 | 3,630 |  | 2.63 | 0.22 |
| 70 | Ngamiland South | U | 69,370 | 2,350 | 4,890 | 4,320 |  | 3.42 | 0.83 |
| 71 | Ngamiland North | R | 52,820 | 2,550 | 2,130 | 2,190 |  | 4.82 | -0.11 |
| 72 | Chobe | PU | 16,250 | 750 | 1,700 | 990 |  | 4.83 | 4.57 |
| 80 | Ghanzi | R | 32,370 | 3,090 | 1,570 | 2,030 |  | 9.41 | -1.40 |
| 90 | Kgalagadi South | R | 25,260 | 1,680 | 1,400 | 1,420 |  | 6.65 | -0.08 |
| 91 | Kgalagadi North | R | 16,510 | 910 | 1,700 | 1,190 |  | 5.69 | 3.19 |
|  | Botswana (Total) |  | 1,673,580 | 53,320 | 127,720 | 127,720 |  | 3.19 | 0 |

**Supplementary file 1d: Migration metrics by district, 2010-11.**

|  |  |  |  | Number of Migrants | | |  | Metric | |
| --- | --- | --- | --- | --- | --- | --- | --- | --- | --- |
| ID | District | Class | Population | Within | In | Out |  | WDMI | Turnover |
| 1 | Gaborone | C | 232,550 | 850 | 22,250 | 29,540 |  | 0.37 | -3.04 |
| 2 | Francistown | C | 99,580 | 150 | 9,420 | 12,880 |  | 0.15 | -3.36 |
| 3 | Lobatse | T | 28,990 | 60 | 3,150 | 3,790 |  | 0.21 | -2.19 |
| 4 | Selebi Phikwe | T | 49,630 | 60 | 4,830 | 5,260 |  | 0.12 | -0.86 |
| 5 | Orapa | T | 9,230 | 0 | 1,420 | 1,810 |  | 0.00 | -4.05 |
| 6 | Jwaneng | T | 17,910 | 40 | 3,200 | 2,970 |  | 0.22 | 1.30 |
| 7 | Sowa | T | 3,580 | 10 | 870 | 660 |  | 0.28 | 6.23 |
| 10 | Southern | U | 128,210 | 4,230 | 7,940 | 8,210 |  | 3.30 | -0.21 |
| 11 | Barolong | R | 54,390 | 2,120 | 3,810 | 3,340 |  | 3.90 | 0.87 |
| 12 | Ngwaketse West | U | 13,810 | 620 | 990 | 890 |  | 4.49 | 0.66 |
| 20 | South East | U | 83,970 | 1,020 | 9,210 | 5,700 |  | 1.22 | 4.36 |
| 30 | Kweneng | U | 299,620 | 10,270 | 18,180 | 12,380 |  | 3.43 | 1.97 |
| 40 | Kgatleng | U | 90,840 | 2,280 | 5,690 | 5,060 |  | 2.51 | 0.70 |
| 50 | Central Serowe | PU | 178,670 | 8,250 | 11,840 | 10,890 |  | 4.62 | 0.54 |
| 51 | Central Mahalapye | PU | 118,350 | 4,250 | 6,450 | 7,370 |  | 3.59 | -0.77 |
| 52 | Central Bobonong | PU | 71,310 | 3,020 | 5,170 | 4,290 |  | 4.24 | 1.26 |
| 53 | Central Boteti | PU | 57,420 | 3,420 | 3,660 | 3,040 |  | 5.96 | 1.09 |
| 54 | Central Tutume | PU | 146,540 | 5,470 | 9,680 | 9,400 |  | 3.73 | 0.19 |
| 60 | North East | R | 59,800 | 2,050 | 4,750 | 4,060 |  | 3.43 | 1.17 |
| 70 | Ngamiland South | U | 89,710 | 2,980 | 4,460 | 6,210 |  | 3.32 | -1.91 |
| 71 | Ngamiland North | R | 59,780 | 3,060 | 1,770 | 2,520 |  | 5.12 | -1.24 |
| 72 | Chobe | R | 21,830 | 1,000 | 2,590 | 1,940 |  | 4.58 | 3.07 |
| 73 | Okavango Delta | R | 2,080 | 50 | 350 | 110 |  | 2.40 | 13.04 |
| 80 | Ghanzi | R | 42,720 | 3,810 | 2,520 | 1,950 |  | 8.92 | 1.35 |
| 81 | CKGR | R | 230 | 10 | 130 | 60 |  | 4.35 | 43.75 |
| 90 | Kgalagadi South | R | 29,900 | 1,540 | 1,390 | 1,720 |  | 5.15 | -1.09 |
| 91 | Kgalagadi North | U | 20,510 | 1,090 | 1,660 | 1,300 |  | 5.31 | 1.79 |
|  | Botswana (Total) |  | 2,011,160 | 61,710 | 147,380 | 147,380 |  | 3.07 | 0 |
